# A Systematic Review and Independent Patient Data Meta-Analysis of Prophylactic Mesh Augmentation for Incisional Hernia Prevention After Abdominal Aortic Aneurysm Surgery (I-PREVENT-AAA) A Collaborative European Hernia Society Project

**DOI:** 10.1097/SLA.0000000000006684

**Published:** 2025-02-26

**Authors:** Rudolf van den Berg, Floris P.J. Den Hartog, Sara J. Baart, Christina Bali, Miltiadis Matsagkas, Paul M. Bevis, Jonothan J. Earnshaw, Eike S. Debus, Susanne Honig, Frederik Berrevoet, Olivier Detry, Cesare Stabilini, Filip E. Muysoms, Pieter J. Tanis, European Hernia Society prophylactic mesh study group collaborators, Holger Diener

**Affiliations:** * Department of Surgery, Erasmus University Medical Centre, Rotterdam, The Netherlands; † Department of Biostatistics, Erasmus University Medical Centre, Rotterdam, The Netherlands; ‡ Department of Surgery, University Hospital of Ioannina, Ioannina, Greece; § Department of Vascular Surgery, University of Thessaly, Larissa, Greece; ∥ Department of Vascular Surgery, North Bristol NHS Trust, United Kingdom; ¶ Department of Vascular Surgery, Gloucestershire Hospitals NHS Foundation Trust, UK; # Department of Vascular Medicine, University Medical Center Hamburg-Eppendorf University Heart & Vascular Center, Eppendorf, Hamburg, Germany; ** Department of Vascular Surgery, Hospital Robert Schuman Kirchberg Hospital, Luxembourg; †† Department of General and HPB Surgery and Liver Transplantation, Ghent University Hospital, Ghent, Belgium; ‡‡ Department of Abdominal Surgery and Transplantation, Division of Abdominal Wall Surgery, CHU Liege, University of Liege, Liege, Belgium; §§ Department of Surgical Sciences, University of Genoa, Genoa, Italy; ∥∥ Department of Surgery, AZ Maria Middelares Hospital, Ghent, Belgium

**Keywords:** incisional hernia, prevention, surgical mesh, suture techniques, aortic aneurysm

## Abstract

**Objective:**

To analyze the effectiveness of prophylactic mesh augmentation (PMA) of the abdominal wall following open aortic aneurysm repair as compared to primary sutured (PS) closure in preventing incisional hernia (IH) formation by performing an individual patient-data meta-analysis (IPDMA).

**Background:**

IH is a prevalent complication after abdominal surgery, especially in high-risk groups. PMA of the abdominal wall has been studied as a preventive measure for IH formation, but strong recommendations are lacking.

**Methods:**

A systematic literature search was conducted till September 23, 2024, to identify randomized controlled trials (RCTs) that compared PMA with PS after open AAA surgery. Lead authors of eligible studies were asked to share individual patient-data. A one-stage analysis was performed, and Cox regression analyses were used to assess time-to-event outcomes.

**Results:**

Five randomized trials with a total of 493 patients were included. Intention to treat analysis revealed that PMA was associated with a significantly lower risk of IH [hazard ratio of 0.25 (95% CI: 0.12–0.50)] as compared with PS closure. Three-year incisional hernia rates were 13.2% and 39.6%, respectively, with a number needed to treat of 3.7. The effect was similar for onlay and retro-rectus PMA. PMA resulted in longer operative time (mean 27 min) and more seroma formation (especially onlay PMA) but did not increase the risk of surgical site infection.

**Conclusions:**

PMA after elective open abdominal aortic aneurysm surgery is proven to be an effective measure to reduce IH formation and should be considered in future guidelines as a standard of care.

Incisional hernia (IH) is a ventral abdominal wall hernia that occurs at the site of a prior surgical incision, presenting primarily with bulging, which enlarges with heightened intra-abdominal pressure.^1^ Typically, symptoms of IH manifest around 2 years postsurgery, posing risks such as bowel obstruction or strangulation necessitating emergency surgery, potentially involving intestinal resection and ostomy formation.^2,3^ The condition can severely affect patients’ quality of life and body image.^4^ Given its prevalence and associated costs, IH repair has notable economic implications,^5,6^ with surgical mesh repair being the definitive treatment.^7^

Patients undergoing elective midline laparotomies face an average 30% risk of IH development,^8^ and this risk can increase to nearly 70% in high-risk groups such as patients undergoing open repair of an abdominal aortic aneurysm (AAA)^9^ or following emergency surgeries.^10,11^ Patients with AAA may have an underlying connective tissue disorder, possibly predisposing them to IH development.^12^ Various strategies, including diverse incision techniques, suture methods, and prophylactic mesh augmentation (PMA) have been explored to prevent IH formation.^13,14^ Recent meta-analyses suggest that PMA during midline laparotomy closure is effective, and cost-efficient in high-risk populations.^15–17^ Nonetheless, strong recommendations remain tentative, mainly due to the limitations of individual trials.^18^

The European Society for Vascular Surgery guidelines suggest considering PMA following open AAA repair, albeit with a class IIa recommendation based on level A evidence.^19^ This recommendation is based on a meta-analysis published in 2018.^20^ Outcomes of the AIDA trial^21^ and long-term outcomes from pivotal RCTs like the PRIMAAT and PRIMA trials^22,23^ were not included in this analysis.

Pooling individual participant data (IPD) from various studies offers a robust approach for evaluating treatment effects while enhancing the reliability and generalizability of findings and facilitating analysis of patient subgroups not documented separately in original publications. This present individual patient data meta-analysis (IPDMA) aimed to increase the level of evidence on PMA post-open AAA repair as compared with primary suture (PS) closure by consolidating IPD from relevant RCTs and conducting statistical analyses on aggregated patient-level data. Furthermore, we aimed to identify individuals most likely to benefit from this intervention and to determine the effect of PMA on operative time, abdominal wall complications, and the need for IH repair surgery.

## METHODS AND ANALYSIS

The study protocol was approved by the EHS scientific committee and subsequently a detailed description of the study was published.^24^ The study adheres to the PRISMA and PRISMA-IPD guidelines and the recommendations by Riley et al.^25–27^ Secure data transfer methods were developed in collaboration with the Erasmus MC, adhering to European data-sharing regulations.

### Literature Search and Selection Criteria

A literature search was performed in Cochrane Central Register of Controlled Trials (CENTRAL), Embase MEDLINE Ovid, Web of Science Core Collection, and Google Scholar, with the last search performed on September 23, 2024. The search strategy was defined together with a professional and experienced librarian. The complete search terms are provided in the supplementary files (Supplemental A, Supplemental Digital Content 1, http://links.lww.com/SLA/F422).

Eligible studies were RCTs comparing PMA with PS after midline laparotomy, including open AAA repair, and reporting IH incidence. Nonrandomized studies were excluded. Individual patients not undergoing open AAA repair, or those with previous hernia repair with mesh were excluded.

### Data Procurement

Corresponding authors of identified studies were contacted to obtain anonymized data sets. Data delivery agreements were drafted by both parties, and anonymized data sets and related data dictionaries were securely transferred to Erasmus MC, for use only as agreed on in the data delivery agreement﻿.

### Data Processing and Validation

The following trial-related data were extracted: funding source, trial design, inclusion and exclusion criteria, sample size, number of intervention arms, study period, number of sites, length of follow-up, and modalities used for the diagnosis of IH. Furthermore, the number of randomized participants, participants lost to follow-up, participant characteristics, treatment characteristics, and studied outcomes were extracted. Data sets of the individual trials were harmonized and compared with original publications. The balancing of baseline characteristics in each treatment arm was evaluated, as well as the extent to which all randomized participants were included in the study analyses. The study authors were consulted in case of any discrepancies.

### Study Quality Assessment

Two investigators (R.v.d.B. and F.P.J.d.H.) independently evaluated the risk of bias using the Cochrane ROB tool, resolving disagreements through discussion. Authors involved in any of the included trials did not extract data or assess the risk of bias in those trials. The Cochrane ROB tool considers 5 domains of bias: randomization, deviations from intended interventions, missing outcome data, measurement of the outcome, and selection of the reported results, all rated with﻿ low, some, or high concerns.

### Outcome Parameters and Statistical Analysis

The primary outcome parameter was the IH rate, analyzed with a one-stage meta-analysis. A time-to-event approach with cox-regression analysis using the outcome of randomization as an independent variable and the trial identifier as a cluster term was used, thereby accounting for clustering at the randomization level. Hazard Ratios (HR, 95% CI) were used to document effect sizes.^28^ The Cox proportional hazards assumption was assessed using the Schoenfeld residuals.

Clinically relevant subgroups were defined pre-analysis based on gender, age, BMI, American Society of Anesthesiologists (ASA) classification, smoking status, and incision length. These factors were hypothesized to modify the effect of PMA on IH formation and were therefore﻿ analyzed separately. The proportional hazards assumptions in these subgroup analyses were﻿ assessed﻿ using the Schoenfeld residuals.

Binary secondary outcomes were surgical-site infection (SSI), seroma formation, fascial dehiscence (FD), and IH repair surgery. These binary outcomes were analyzed using logistic regression models accounting for clustering on the trial level. Total operative time and closure time were analyzed with linear regression models after transforming the data on the log scale. A binary IH outcome measure was analyzed to compare results from published standard aggregated meta-analyses. Sensitivity analyses were conducted to analyze the effect of a single trial on the pooled outcome. Complete data was present and therefore no missing data imputation was necessary. Statistical analyses were performed using R studio, version 4.4.1,^29^ with a 2-sided significance set at *P*<0.05.

## RESULTS

Overall, 369 articles were identified by the literature search (Supplemental B, Supplemental Digital Content 1, http://links.lww.com/SLA/F422). After the exclusion of duplicates, the titles and abstracts of 256 records were screened. Twenty-one full-text articles were assessed for eligibility. Seven articles reporting RCTs on PMA for laparotomy closure^21–23,30–33^ were found. Two of those articles comprised updates with long-term follow-up, resulting in inclusion of 5 RCTs in the present analysis (Table 1 and Supplemental C, Supplemental Digital Content 1, http://links.lww.com/SLA/F422). Investigators of all studies agreed to share their IPD, enabling a one-stage IPDMA.

**TABLE 1:** Characteristics of Included Randomized Controlled Trials on Prophylactic Mesh Reinforcement During Laparotomy Closure

Author	Month and year of enrollment	Mesh group (n)	Control group (n)	Follow-up months (Q_1_, median, Q_3_)	Mesh placement	Hernia examination
Bevis et al^33^	From 11-2003 till 03-2007	40	45	36 (23, 24, 49)	Retro-rectus	Ultrasound
Bali et al^32^	From 09-2007 till 03-2009	20	20	36 (36, 36, 36)	Onlay	CT
Honig et al^21^	From 02-2011 till 07-2013	32	72	24 (12, 12, 31)	Onlay	Ultrasound
Dewulf et al^23^	From 02-2009 till 01-2013	56	58	60 (40, 49, 89)	Retro-rectus	Clinical examination, ultrasound and CT
Van den dop et al^22^	From 03-2009 till 12-2012	113	37	136 (27, 44, 141)	Onlay and retro-rectus mesh placement	Clinical examination, ultrasound and CT

Detailed description of the inclusion and exclusion criteria of the individual studies as well as the technical details of PMA and PS are documented in the supplementary files (Supplemental C, Supplemental Digital Content 1, http://links.lww.com/SLA/F422). Three trials randomized between a single PMA technique and a single PS technique, while the PRIMA trial randomized patients to either onlay PMA, retro-rectus PMA, or PS, and the AIDA trial randomized patients to onlay PMA, PS with long-term absorbable sutures, or PS with extra long-term absorbable sutures. Four of the published RCTs used a synthetic polypropylene mesh^21–25,33,34^ and the study by Bali et al reported the use of a bovine pericardium mesh^32^ (Supplemental C, Supplemental Digital Content 1, http://links.lww.com/SLA/F422).

### Risk-of-bias Assessment

Risk-of-bias assessment using the ROB-2 tool and a detailed assessment is available in the supplementary files (Supplemental D and E, Supplemental Digital Content 1, http://links.lww.com/SLA/F422). The study by van den Dop and colleagues had a low risk of bias across all criteria. In the trial by Dewulf and colleagues, abdominal wall surgeons closed the abdominal wall, indicating high-quality standards, but no standard radiologic outcome assessment was used. Detection bias was identified in Bevis et al‘s study due to non-blinded outcome assessors. Bali and colleagues' study used standardized CT assessment for all patients, albeit without blinding assessors. Bali and colleagues' trial raised concerns regarding reporting bias as it lacked prospective registration. Although the trials by Dewulf colleagues and van den Dop and colleagues received partial funding from mesh and glue manufacturers, no conflicts of interest were reported.

### Study Population

From the 5 identified RCTs, a total of 493 patients could be included. Baseline characteristics of the 5 individual study populations as well as the combined population are summarized in Table 2. Among all patients, 56 were females and 437 males. The pooled mean age was 71 years (95% CI: 70.4–71.7), the pooled mean BMI was 26.4 (95% CI: 26.0–26.8), 15% had COPD, and 13% had diabetes. In total, 261 patients were randomized to PMA and 232 to PS.

**TABLE 2:** Baseline and Treatment Characteristics of the Patients Who Underwent Open AAA Repair From the 5 Included Trials

Characteristics	Overall, N=493	Honig et al,^21^ N=104	Bali et al,^32^ N=40	Bevis et al,^33^ N=85	Van den dop et al,^22^ N=150	Dewulf et al,^23^ N=114	*P*
Age, n (%)*	71 (8)	69 (8)	74 (6)	73 (7)	70 (7)	72 (8)	<0.001†
ASA score, n (%)​‡							
1	17 (5)	0	0 (NA)	0 (NA)	7 (5.1)	10 (8.9)	—
2	189 (54)	41 (40)	0 (NA)	0 (NA)	79 (58)	69 (62)	—
3	143 (41)	61 (59)	0 (NA)	0 (NA)	49 (36)	33 (29)	—
4	3 (0.1)	1 (1.0)	0 (NA)	0 (NA)	2 (1.5)	0	—
Sex, n (%)‡	—	—	—	—	—	—	0.2§
F	56 (11)	10 (10)	4 (10)	8 (10)	25 (17)	9 (8)	—
M	437 (89)	94 (90)	36 (90)	77 (91)	125 (83)	105 (92)	—
BMI*	26.4 (3.9)	27.0 (4.0)	24.6 (2.4)	NA (NA)	26.8 (4.3)	26.0 (3.7)	0.004§
Diabetes, n (%)‡	62 (13)	4 (4)	10 (25)	10 (12)	19 (13)	19 (17)	0.026∥
Connective tissue disorder, n (%)‡	11 (6)	9 (8.9)	0 (NA)	2 (2)	0 (NA)	0 (NA)	0.110§
COPD, n (%)‡	74 (14.6)	16 (15)	17 (43)	7 (8.5)	0 (NA)	34 (31)	<0.001§
Active smoking, n (%)‡	194 (39)	49 (47)	0 (NA)	23 (27)	53 (42)	69 (64)	<0.001§
Type of closure, n (%)‡							
Primary suturing	232 (47)	72 (69)	20 (50)	45 (53)	37 (25)	58 (51)	—
Onlay mesh	113 (23)	32 (31)	20 (50)	0	61 (41)	0	—
Retro-rectus mesh	148 (30)	0	0	40 (47)	52 (35)	56 (49)	—
Incision length^¶^ *	28 (7)	26 (8)	NA (NA)	NA (NA)	30 (8)	28 (4)	<0.001†

* Mean (SD).

† Kruskal-Wallis rank sum test.

‡ n (%).

§ Fisher exact test.

∥ Pearson χ^2^ test.

^¶^ Measured ﻿in centimeters﻿

### Primary outcome

**FIGURE 1:**
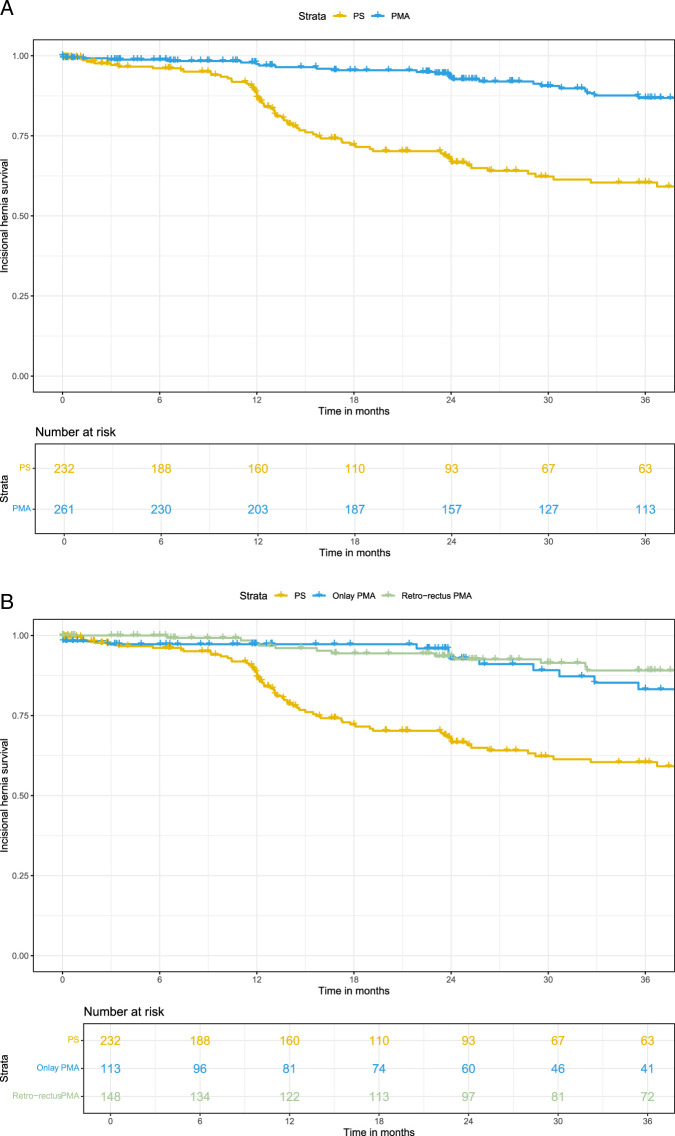
A and B, Kaplan-Meier curves for patients treated with primary closure or prophylactic mesh augmentation (A) and prophylactic mesh augmentation stratified for onlay and retro-rectus mesh positions (B).

The primary outcome was assessed with a median follow-up of 24 months (IQR 12–40). IH was diagnosed in 34 of 261 patients in the PMA group and in 78 of 232 patients in the PS group. According to intention-to-treat analysis, the use of PMA was associated with a significantly lower risk of IH compared with PS (adjusted HR 0.25, 95% CI: 0.12–0.50, *P*<0.0001) (Fig. 1). The 3-year IH rate was 13% after PMA, and 40% after PS, corresponding with an absolute 27% risk reduction and a number needed to treat (NNT) of 3.7. The proportional hazard assumption was satisfied in both onlay and retro-rectus groups. Schoenfeld residuals can be found in Supplementary Figures F and G, Supplemental Digital Content 1, http://links.lww.com/SLA/F422, and survival curves of the individual trial populations in supplementary figure H, Supplemental Digital Content 1, http://links.lww.com/SLA/F422.

**FIGURE 2:**
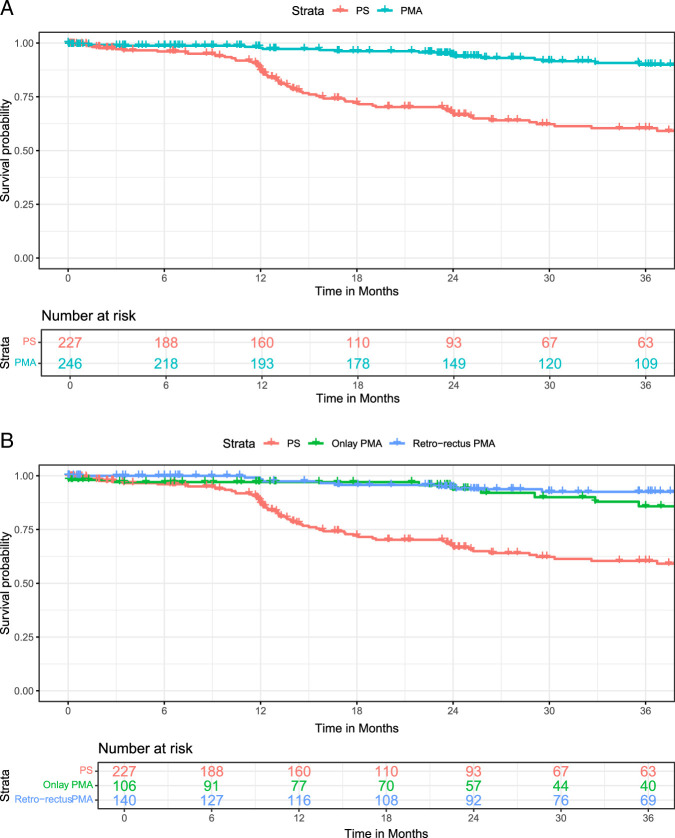
A, B: Kaplan-Meier curves for the per-protocol analysis of patients treated with primary closure or prophylactic mesh augmentation (A) and prophylactic mesh augmentation stratified for onlay and retro-rectus mesh positions (B).

Due to deviations from the intended intervention, a per-protocol analysis was conducted. In the PRIMA trial, 15 patients did not receive a mesh after randomization, 7 in the onlay PMA and 8 in the retro-rectus PMA group. In the AIDA trial, 5 other deviations arose: 4 patients due to the abdominal incision length of the laparotomy being over 30 cm and 1 other patient because of an intraoperative death due to myocardial infarction. No further deviations were noted. Per-protocol analysis showed a similar IH reduction using PMA (HR 0.20, 95% CI: 0.10–0.39) as compared with PS, with 3-year IH rates of 10% and 40%, respectively (NNT 3.4). Among patients who received PMA, no significant differences in IH rate were found between onlay and retro-rectus PMA (16/106 and 11/140, respectively, HR 1.81; 95% CI: 0.79–4.17) (Fig. 2).

### Sensitivity Analysis for the Primary outcome

Sensitivity analyses showed that all combination of trials were found to result in a reduction of the IH rate with an adjusted HR lower than 0.32 (Supplemental I, Supplemental Digital Content 1, http://links.lww.com/SLA/F422). The largest impact on the HR was noted when excluding the PRIMA and PRIMAAT trials.

### Subgroup Analysis for the Primary outcome

**FIGURE 3:**
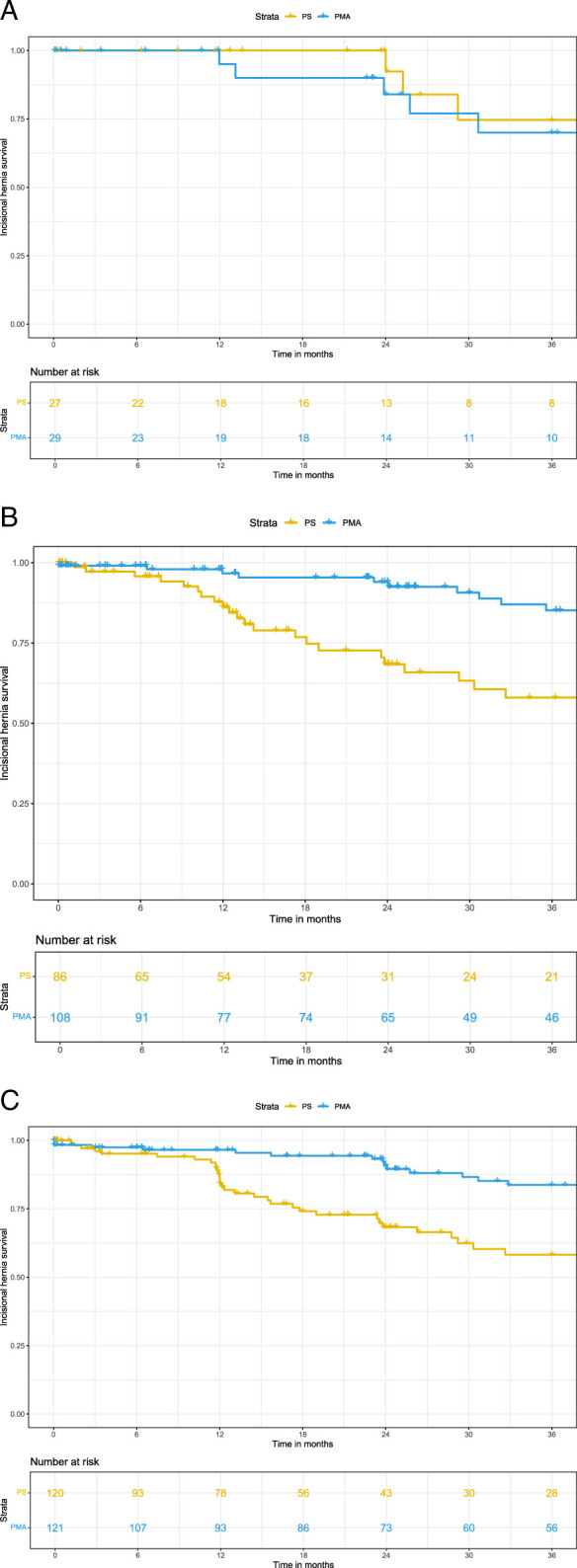
A–C, Kaplan-Meier curves for patients treated with primary closure or prophylactic mesh augmentation in women (A), smokers (B), and patients with a BMI >25 (C).

**FIGURE 4:**
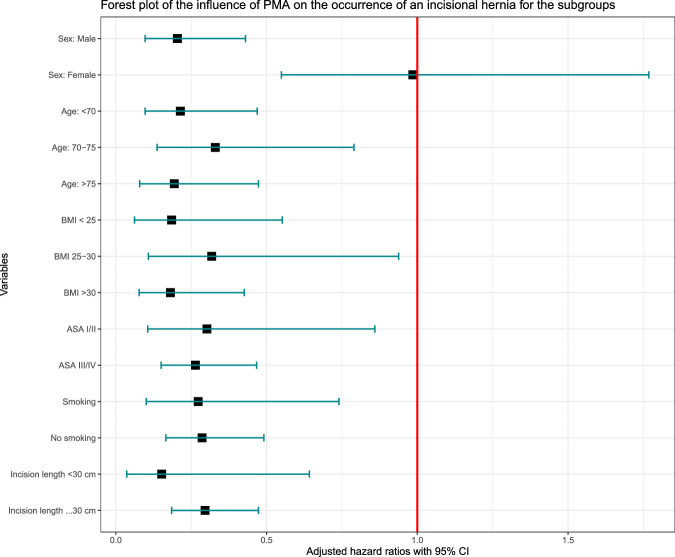
Forest plot on the effect of prophylactic mesh augmentation on the occurrence of an incisional hernia for the predefined subgroups.

Subgroup analysis showed that PMA after open AAA surgery resulted in a significantly lower IH rate across all age categories, ASA scores, BMI categories, incision length categories and smokers or non-smokers as compared with PS. Only in the small subgroup of 56 females, no significant reduction in IH rate by PMA could be demonstrated (Figs. 3 and 4). The proportional hazard assumptions were satisfied in all subgroups. Figures of the Schoenfeld residuals can be found in Supplementary Figures J–L, Supplemental Digital Content 1, http://links.lww.com/SLA/F422.

### Secondary outcomes

The cumulative rates of SSI and seroma were 1.9% and 0.5% for PS versus 2.2% and 11.5% for onlay PMA, and 3.3% and 2.2% for retro-rectus PMA, respectively. In the PS group, no postoperative FDs were seen while FD occurred in 3.2% and 3.8% of the patients in the onlay and retro-rectus groups (Table 3). The binary IH outcome measure occurred less frequently in the PMA group compared with the PS group (OR: 0.17; 95% CI: 0.09–0.28, *P*<0.0001) with IH repair surgery conducted less frequently in the PMA group compared with the PS group (OR: 0.18; 95% CI: 0.04–0.60, *P*=0.011). Closure time and total operation time were significantly longer in the PMA groups compared with the PS group. Median operation time for retro-rectus PMA was 189 minutes (IQR: 155–240), and 195 minutes (IQR: 160–225) for onlay PMA.

**TABLE 3:** Secondary Outcomes Across All Trials

	Event proportions						
	PMA			Median (IQR)			
Outcome	Onlay	Retro-rectus	PS	PMA	PS	Amount of trials reporting the outcome	*P*
SSI	2/93	3/92	3/154	—	—	3^*^, ^†^, ^‡^	0.650^§^
Seroma	13/113	2/92	1/174	—	—	4^*^, ^‖^, ^†^, ^‡^	0.012^§^
IH occurrence	18/113	16/148	78/232	—	—	5^*^ ^‖^ ^†^ ^¶^ ^‡^	<0.001^§^
Reoperation for IH	2/88	1/141	12/207	—	—	4^*^, ^†^, ^¶^. ^‡^	0.011^§^
Operation time ^#^	—	—	—	192 (155–240)	165 (131–199)	4^‖^, ^†^, ^¶^, ^‡^	0.002^**^
Closure time ^#^	—	—	—	42 (25–55)	20 (18–29)	2^†^, ^¶^	<0.001^**^

^*^ AIDA.

^†^ Bevis.

^‡^ PRIMA.

^§^ *P* value stems from a logistic regression model comparing all PMA patients to PS.

^‖^ Bali.

^¶^ PRIMAAT.

^#^ Time in minutes.

^**^ *P* value stems from a linear regression model with a logistic transformation of the time scale.

## DISCUSSION

This independent patient data meta-analysis based on 5 randomized trials shows that the use of PMA after midline laparotomy for open AAA repair surgery significantly decreases the IH rate as compared with PS. The intention-to-treat analysis showed a hazard-ratio of 0.25 (95% CI: 0.12–0.50), with a 27% absolute IH rate reduction at 3 years, corresponding with a NNT of 3.7. PMA was proven to be effective across all subgroups, although statistical significance was not reached for female patients. Also, onlay and retro-rectus PMA were similarly effective. PMA slightly increased the operative time and onlay PMA was associated with seroma formation. However, no increased risk of SSI was observed, and significantly ﻿fewer IH repair operations were conducted.

The most recently conducted meta-analysis by Indrakasuma and colleagues reported a risk-ratio of 0.27 (95% CI: 0.11–0.68). After adding an additional trial and updated follow-up of 2 trials, our IPDMA resulted in a more precise odds-ratio of 0.17 (95% CI: 0.09–0.28, *P*<0.0001) for IH reduction if using the same statistical analyses as Indrakasuma et al.^20^ In addition, we were able to demonstrate significantly less IH-repairs after PMA for the first time. By using IPD in the present study, we were able to perform time-to-event analysis with HRs and subgroup analysis for onlay versus retro-rectus PMA, as well as for all clinically relevant entities. Therefore, the results of the present study add significantly to the body of evidence since previously conducted meta-analyses.

Obtaining large sample sizes for new trials will be challenging due to the increasing popularity of endovascular AAA repair, with a corresponding decreasing trend for open repair.^35^ The European society of vascular surgery recommends endovascular AAA repair in most patients with suitable anatomy and reasonable life expectancy as the preferred treatment modality.^19^ In the United States, open AAA repair decreased from 20,533 in 2003 to 4916 in 2014.^34^ Therefore, this IPDMA likely included all available data for the foreseeable future as no planned or ongoing studies have been published on Clinicaltrials.gov’s register as of October 2024.

Onlay PMA increased seroma formation, although generally not posing a clinically relevant problem. However, an increased risk of infectious complications has been described after onlay PMA, when compared with retro-rectus PMA.^36^ Retro-rectus mesh may be preferable, but its technical complexity could pose a threshold in performing PMA.^18^ This might be the case for vascular surgeons not trained in retro-rectus dissection. Including onlay PMA in guideline recommendations was based on the opinion that enforcing surgeons to follow only retro-rectus PMA could negatively affect support for PMA in general.^18^ Our IPDMA provides convincing evidence for routine implementation of PMA. The optimal way of PMA should subsequently be discussed in each unit. Given the results of individual trials, retro-rectus PMA would be preferable as in the PRIMAAT trial, where an abdominal wall surgeon performed retro-rectus PMA after the vascular surgeon finished open AAA repair, this resulted in excellent outcomes. For the few open AAA repairs that are performed yearly, it should be feasible to achieve this.

Mesh reinforcement can be improved by combining it with small-bites fascial closure.^13^ The use of small-bites was suggested in the included trials, but no quality control was implemented to ensure implementation of the technique. Another technical element to consider is mesh overlap and fixation. The mesh in the PRIMAAT and AIDA trials was placed with an overlap of 7.5 cm in the retro-rectus area, but in the PRIMA trial, the overlap was smaller and the mesh was fixed with glue instead of stitches. The 2010 Ventral Hernia Working Group recommended a minimal overlap of the mesh of 5 cm during hernia repair.^37^ However, it is questionable if such a large overlap is advisable in PMA.

During 5-year follow-up in the PRIMAAT trial, 21.7% of the patients in the PS group underwent reoperation due to IHs.^30^ Although surgeons may be hesitant to operate on IHs in AAA patients, reoperation rates for IH in the literature after open AAA repair range between 9.3% and 10.4%.^17,20^ Although only 17.4% of patients with an IH reported hernia-related symptoms, these long-term results confirm the substantial rates of reoperation due to IHs when no PMA was performed. Several authors have shown that IHs pose a clinically relevant problem and are associated with a major economic burden.^4–6^ Thus, although our study reports that only 5% to 6% of patients undergo an IH repair, the overall burden of an IH on a patient highlights the significant value of implementing PMA.

This study proves the effectiveness of PMA in the prevention of IHs in a specific high-risk group, which raises the question whether to extrapolate these findings to other high-risk patients. PMA could for example be considered after laparotomy in patients with obesity, in patients with connective tissue disorders or after prior laparotomies. Mainly, the present study should make surgeons aware that PMA is a valid option for abdominal wall closure in patients at increased risk of IH.^38^

Despite generating the highest level of evidence on PMA for laparotomy closure after open AAA repair, this study is also subject to some limitations. First, abdominal wall closure was heterogeneous regarding technique and relevant experience of the surgeons. Furthermore, no quality control was present for closures in any of the trials. This may have increased the external validity of the findings by reflecting routine daily practice on one hand but might have also underestimated the impact of PMA if performed most optimally. Second, only elective cases were included in this study. This limits the extrapolation of these results to all cases of open AAA repair, which is also performed in the emergency setting. Furthermore, female patients were underrepresented, although the direction of treatment effect was similar, and the results are in line with a recent comparative cohort study in a female population.^39^ Finally, secondary endpoints could not be evaluated for all included trials, which might have resulted in underpowered analyses.

Current guidelines strongly advocate for a 4:1 ratio of suture length to wound length following open AAA repair, but lack a definitive recommendation regarding the use of PMA.^18,19^ A decisive recommendation in forthcoming guidelines, supported by extensive follow-up data and our IPDMA, should lead to more widespread adoption of PMA during open AAA repair surgery.

## CONCLUSIONS

The current individual patient data meta-analysis shows that PMA of the abdominal wall after open midline laparotomy for the treatment of an AAA effectively and substantially decreases the rate of IH in the overall population and all subgroups except for the minority of female patients. This study has bundled all available randomized trial data resulting in the highest grade of evidence for PMA﻿ after open ﻿AAA-surgery to prevent the formation of an IH. For the foreseeable future, similar RCTs are unlikely to be conducted given the shift toward endovascular AAA repair, for which reason conclusions based on this study are unlikely to change.

## Supplementary Material
