# Supplementary material for: A Systematic Review and Independent Patient Data Meta-Analysis of Prophylactic Mesh Augmentation for Incisional Hernia Prevention After Abdominal Aortic Aneurysm Surgery (I-PREVENT-AAA) A Collaborative European Hernia Society Project
Source: Ann Surg. 2025 Feb 26;283(1):82–91. doi: 10.1097/SLA.0000000000006684 (PMC12695395; doi:10.1097/SLA.0000000000006684)

**Supplementary files**

**Suppl. A: Search terms for databases Medline, Embase, Web of Science, Cochrane Central and Google Scholar**

**medline ALL Ovid**

(Aortic Aneurysm, Abdominal / OR Aortic Aneurysm / OR ((Aneurysm /) AND Aorta, Abdominal /) OR ((aort* ADJ3 aneurysm*) OR aaa).ab,ti.) AND (Surgical Mesh / OR (mesh* OR dynamesh* OR vitamesh* OR surgimesh*).ab,ti.) AND (exp Preventive Health Services / OR prevention.fx. OR (prevent* OR prophyla* OR augment* OR reinforce*).ab,ti.)

**embase.com**

('abdominal aortic aneurysm'/exp OR 'aortic aneurysm'/de OR ((aneurysm/de OR 'aneurysm surgery'/de) AND 'abdominal aorta'/de) OR ((aort* NEAR/3 aneurysm*) OR aaa):Ab,ti) AND ('surgical mesh'/exp OR (mesh* OR dynamesh* OR vitamesh* OR surgimesh*):ab,ti) AND (prophylaxis/de OR prevention/de OR prevention:lnk OR (prevent* OR prophyla* OR augment* OR reinforce*):ab,ti)

**Web of science**

TS=((((aort* NEAR/2 aneurysm*) OR aaa)) AND ((mesh* OR dynamesh* OR vitamesh* OR surgimesh*)) AND ((prevent* OR prophyla* OR augment* OR reinforce*)))

**Cochrane CENTRAL**

(((aort* NEAR/3 aneurysm*) OR aaa):Ab,ti) AND ((mesh* OR dynamesh* OR vitamesh* OR surgimesh*):ab,ti) AND ((prevent* OR prophyla* OR augment* OR reinforce*):ab,ti)

**Google scholar**

"aortic|aorta aneurysm|aneurysms" mesh|dynamesh|vitamesh|surgimesh preventive|prevention|prophylaxis|prophylactic|augmentation|reinforcement

**Suppl. B: Flow chart of the included studies**

Identification

Screening

Included

Records identified from:

Databases (n = 369)

Records removed before screening:

Duplicate records (n = 113)

Records screened

(

= 256)

n

Records excluded

(

n

= 235)

Reports sought for retrieval

(

n

= 21)

Reports not retrieved

(

n =

0)

Reports assessed for eligibility

(

n

= 21)

Reports excluded:

Different follow-up durations of

the same RCT (n = 12)

Substudy of the sample RCT (n = 2)

Protocol paper (n = 2)

New studies included in review

(

n =

5)

**Suppl.C: Table on exclusion criteria, primary and mesh closure**

| Trial | Inclusion criteria | Exclusion criteria | Primary closure | Mesh closure |
| --- | --- | --- | --- | --- |
| Bali et al. | - Listed for an elective open AAA repair  - Over 18 years  - Median laparotomy | -Previous abdominal surgery  -Steroid usage  -Immunosupressive drug usage | -Routine mass closure in a running polydioxanone loop suture Νο. 1.  - The rule of 4:1 suture to wound length ratio was applied | - A biological mesh derived from processed bovine pericardium  - 4 cm overlap over the sutured fascia  - Non absorbable suture mesh fastening  - Small incisions in mesh to prevent serum accumulation  - Suction drains in the subcutaneous space |
| Bevis et al. | - Listed for an elective open infra-renal AAA repair  - Over 18 years  - Median laparotomy | No exclusion criteria | -Routine mass closure of the abdominal wound using the operating surgeon's preferred non-absorbable monofilament suture material.  -The rule of 4 : 1 suture to wound length ratio was applied | -15 x 15 cm povidone-iodine-soaked polypropylene mesh  - fastened to the posterior rectus sheath with four 2/0 polypropylene tacking sutures, one superior, one inferior, and two laterally where the two mesh parts overlapped  - The anterior rectus sheath was then closed with the same suture used in the control group for mass closure  -The skin was stitched secured using the surgeon's preferred suture material |
| Honig et al. | - Listed for an elective open AAA repair  - Over 18 years  - Median laparotomy | -Previous midline incision  -Emergency AAA surgery  -Life expectancy <24 months  -Immunosupressive treatment  -Radiotherapy on the treated region within the last 2 months  -Participating in other investigational drugs  -Pregnancy  -Mental or social reasons affecting study requirements  -Incision > 30 cm | -Fascial closure was achieved by a long-term absorbable synthetic monofilament suture made of polydioxane (size USP 1, needle HRT 48, 150 cm loop  --The rule of 4 : 1 suture to wound length ratio was applied | - A polydioxane-based long-term absorbable synthetic monofilament suture  - The anterior rectus fascia and subcutaneous fat tissue were separated by a 10 cm wide space  - monofilament, light-weight, large-pored polypropylene mesh was then placed ventrally of the anterior rectus fascia, 5 cm to the right and left and 2.5 cm cranially and caudally overlapping the incision  - The mesh was anchored to the aponeurotic surface using interrupted stitches of a long-term absorbable suture |
| Dewulf et al. | - Listed for an elective open AAA repair  - Over 18 years  - Median laparotomy | -Emergency surgery  -Presence of mesh from previous abdominal hernia repair  -ASA>4  -Unavailability of the abdominal surgeon to attend the operation | -The laparotomy was closed with a slowly absorbable running polydioxanone suture in a single layer  - The rule of 4 : 1 suture to wound length ratio was applied | - In a retromuscular position, a large pore, partially absorbable, and light-weight polypropylene mesh with a width of 7.5 cm was used  - On both sides of the midline, the plane behind the rectus muscles, anterior to the posterior rectus fascia, was dissected for at least 3 cm. A slowly absorbable polydioxanone running suture was used to close the posterior rectus fascia and the peritoneum on the midline  - A mesh large enough to overlap by 3 cm in all directions was cut to size and placed in the retromuscular plane. The mesh was attached to the posterior rectus fascia with rapid absorbable polyglycolic acid sutures . The anterior rectus fascia was closed with a slowly absorbable running polydioxanone suture above the mesh |
| VAN DEN DOP et al. | - Listed for an elective open AAA repair or laparotomy with a BMI >27 kg/m^2^  - Over 18 years  - Median laparotomy | -Emergency surgery -IH in medical history -Included in other trials  -Life expectancy <24 months  -Pregnancy  -Immunosupressive treatment in the last 2 years -Bovine allergy | The midline fascia was closed with running, slowly absorbable sutures (USP 1, needle HRT 48, 150 cm loop) preferably with a loop technique.  -The rule of 4 : 1 suture to wound length ratio was adviced  -[Subcutaneous tissue](https://www.sciencedirect.com/topics/medicine-and-dentistry/subcutaneous-tissue) and skin were closed with sutures preferred by the surgeon | The midline fascia was closed with running, slowly absorbable sutures with a recommended suture length-to-wound length ratio of 4:1 for onlay mesh reinforcement.   For onlay mesh reinforcement:  -an 8-cm-wide anterior plane was formed between the anterior rectus fascia and the subcutis.  -The anterior rectus fascia was wrapped in a lightweight polypropylene mesh with a 3 cm overlap. The mesh was cut to size.  -In the case of an incision longer than 35 cm, two meshes were tied together to achieve a 3 cm overlap. The mesh was glued at its center an edges to the tissue and was fixed in the dissected space with 40 mL of fibrin sealant. -The surgeon's preferred sutures were used to close the subcutaneous tissue and skin.   For retro-rectus mesh reinforcement,  -a posterior plane was created between the posterior rectus sheath and the rectus muscle, as well as a caudal plane to the arcuate line between the peritoneum and the rectus muscle.  -Running, slowly absorbable sutures were used to close the posterior fascial/peritoneal plane, with a recommended suture length-to-wound length ratio of 4:1.  -The posterior rectus fascia was wrapped in a lightweight polypropylene mesh with a 3 cm overlap.  -Mesh adjustments were made in the same manner as described for onlay placement, and the mesh was fixed in the same manner as described for onlay mesh reinforcement.  -The surgeon's preferred sutures were used to close the subcutaneous tissue and skin. |

Suppl. D: Risk of bias plots in the included studies


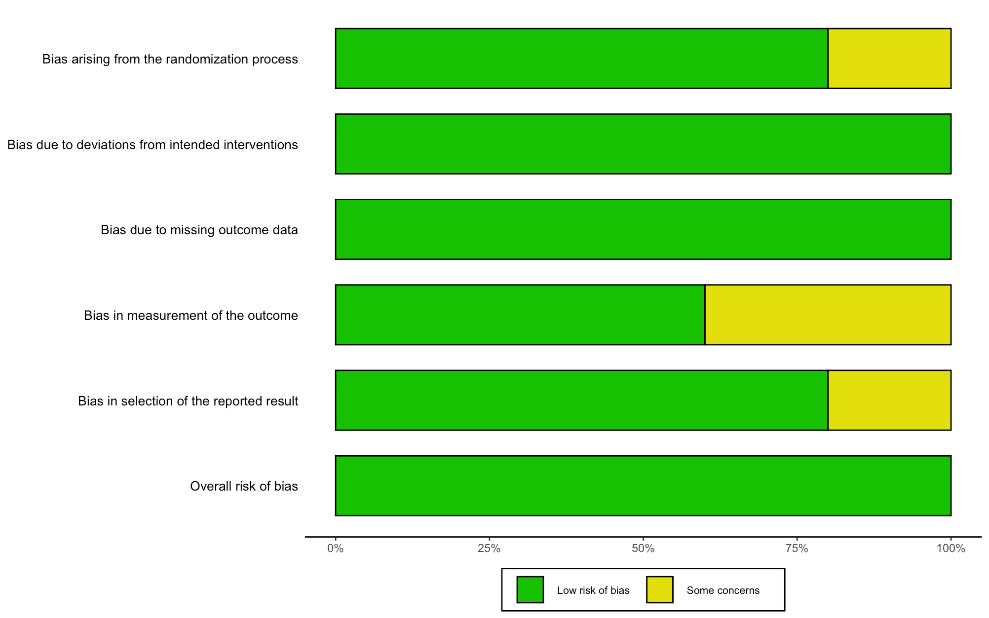


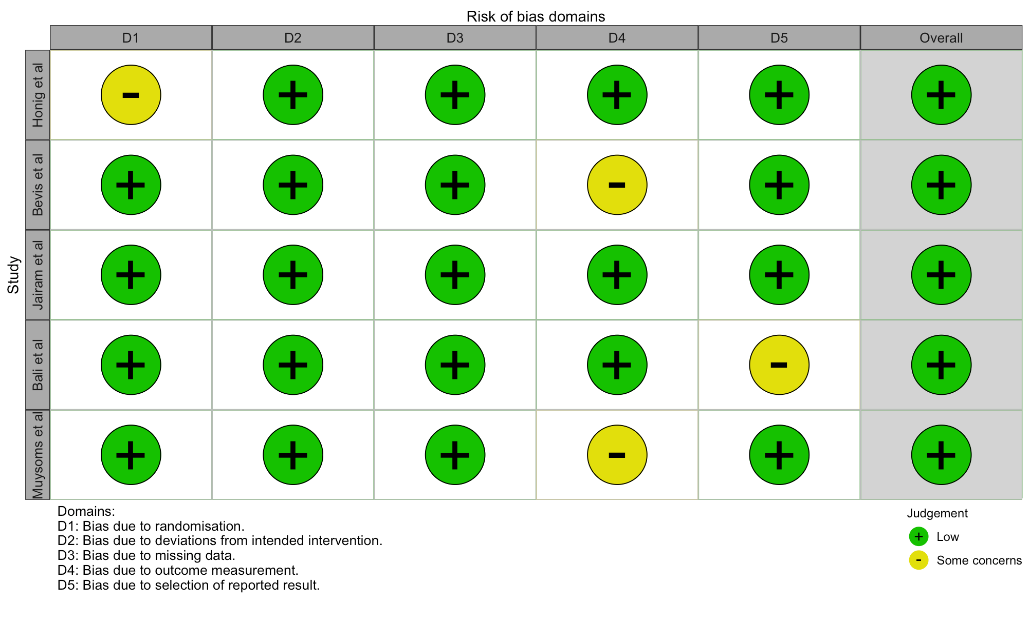


**Suppl. E: Risk of bias assessment of the included trials**

| Study | D1 | D2 | D3 | D4 | D5 | Overall risk of bias |
| --- | --- | --- | --- | --- | --- | --- |
| *Bali et al.* | Low | Low | Low | Low | Some | Low |
| *Bevis et al.* | Low | Low | Low | Some | Low | Low |
| *Honig et al.* | Some | Low | Low | Low | Low | Low |
| *Van den dop et al.* | Low | Low | Low | Low | Low | Low |
| *Muysoms et al.* | Low | Low | Low | Some | Low | Low |

**Suppl. F: Schoenfeld residuals for the overall mesh group**


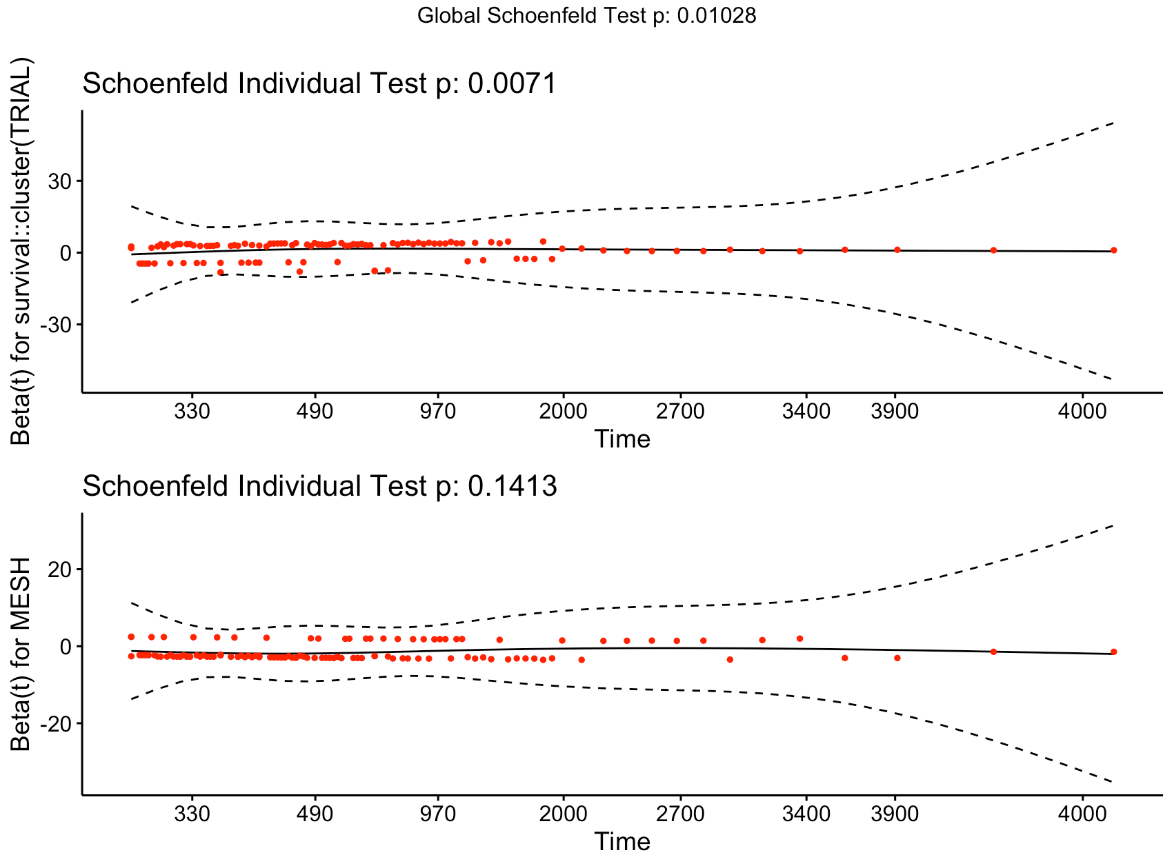


**Suppl. G: Schoenfeld residuals stratified by the type of mesh**


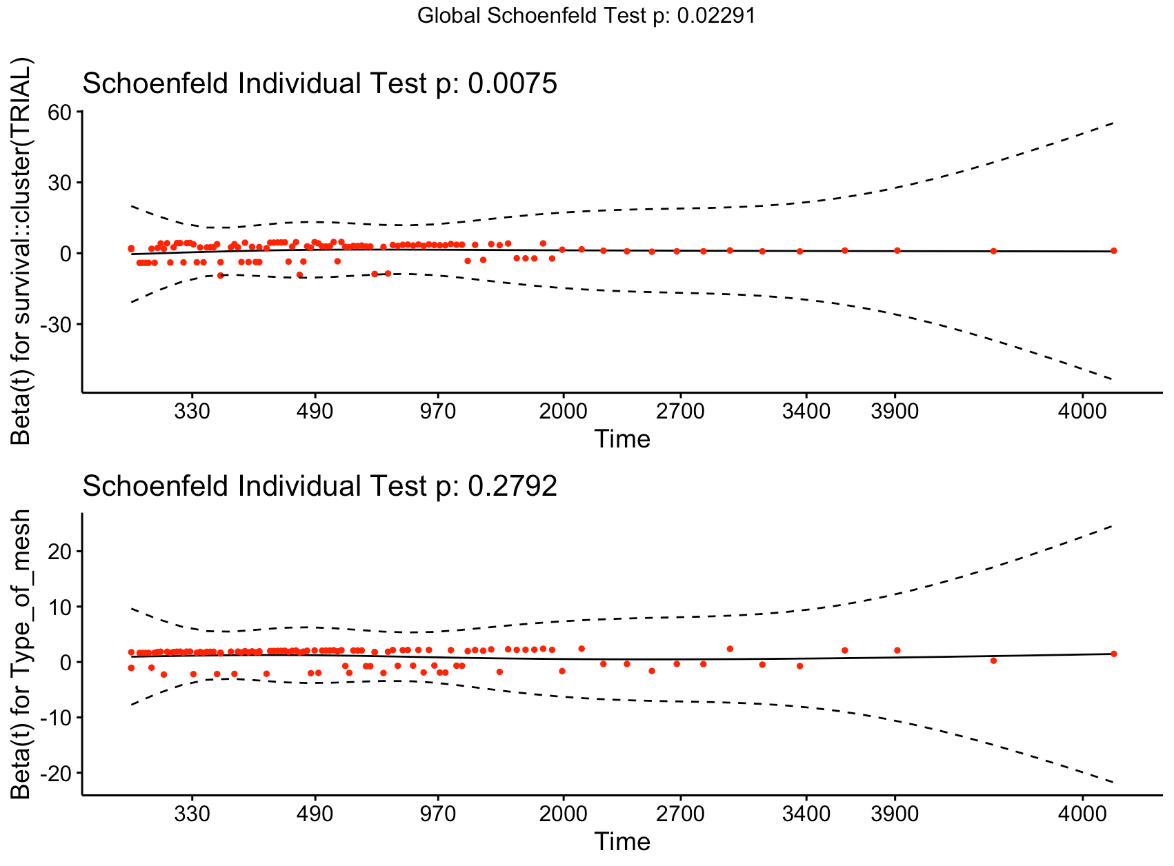


**Suppl. H: Survival curves per trial of the overall population**


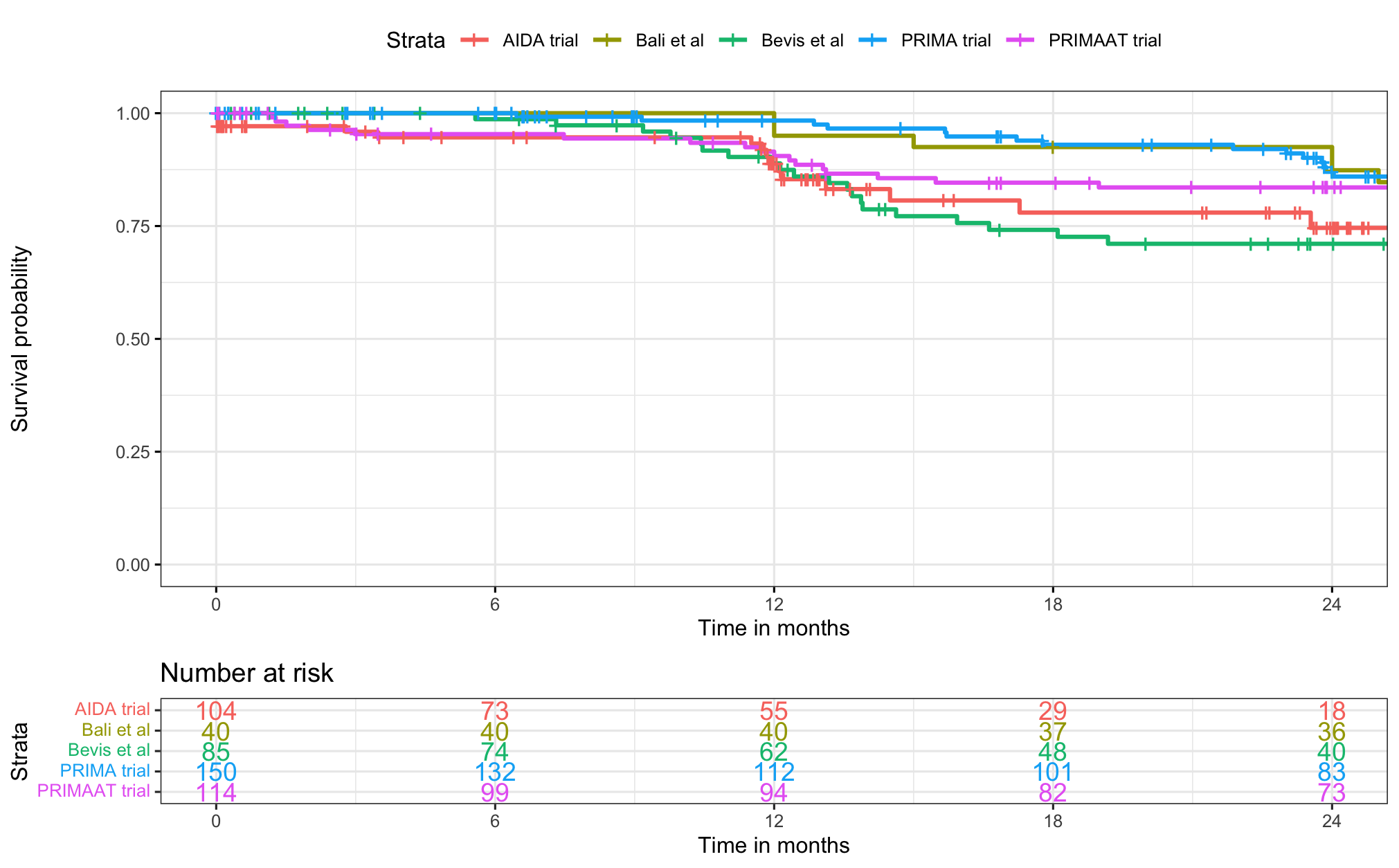


**Suppl. I: Sensitivity analysis with removal of each of the trials from the analysis to assess the influence of specific studies on the results.**

| Removal of which trial from the analysis | HR | 95%CI |
| --- | --- | --- |
| Bali et al. | 0.26 | 0.14-0.50 |
| Bevis et al. | 0.26 | 0.12-0.58 |
| Honig et al. | 0.24 | 0.10-0.54 |
| Van den Dop et al. | 0.11 | 0.03-0.42 |
| Dewulf et al. | 0.32 | 0.23-0.44 |

**Suppl. J: Schoenfeld residuals for the subgroup with a BMI over 25**

Schoenfeld residuals for the subgroup with a BMI > 25


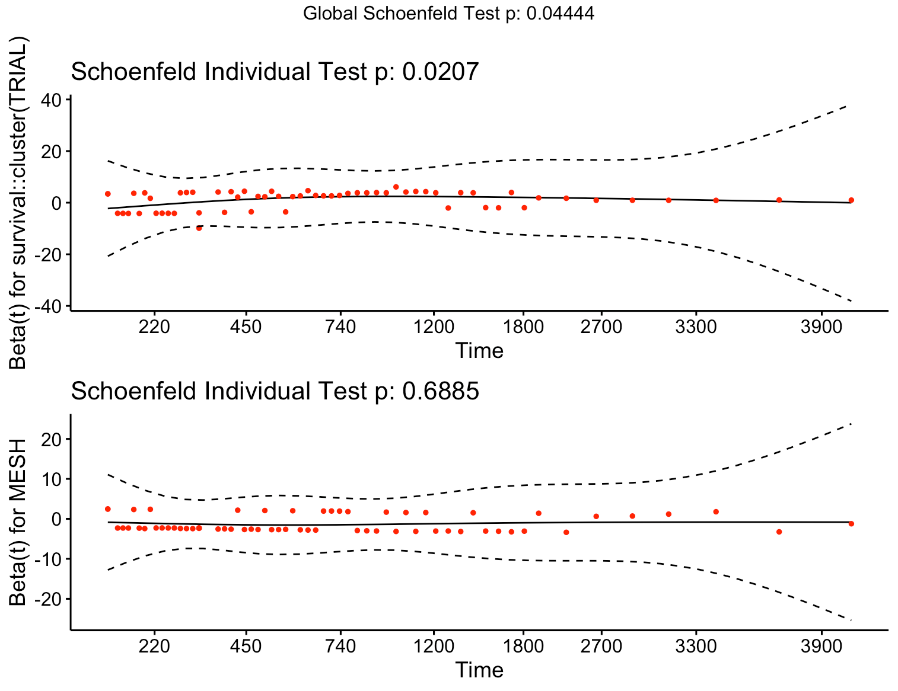


**Suppl. K: Schoenfeld residuals for the subgroup of females**


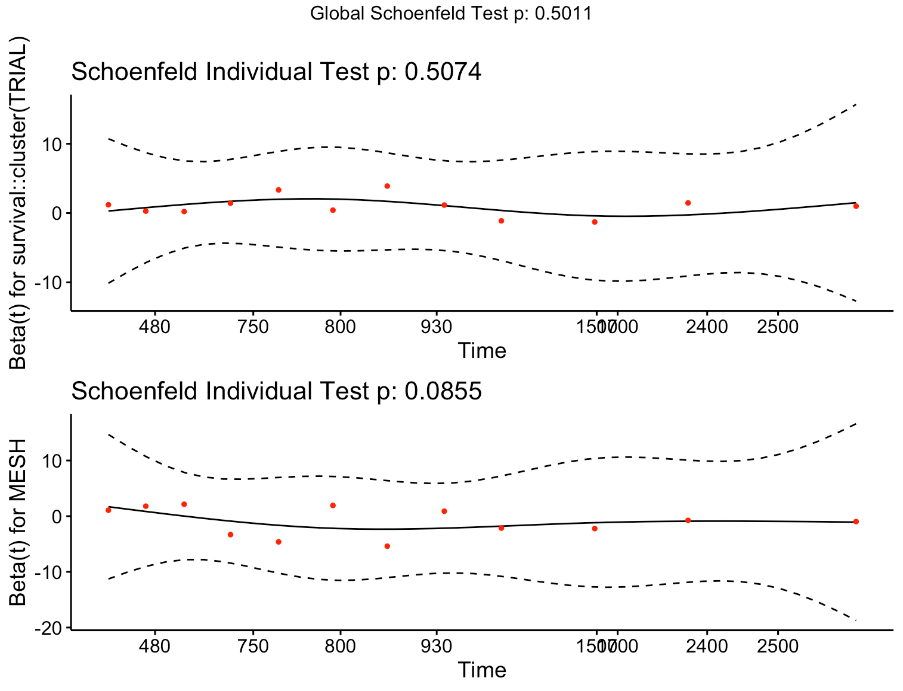


**Suppl. L: Schoenfeld residuals for the subgroup of smokers**


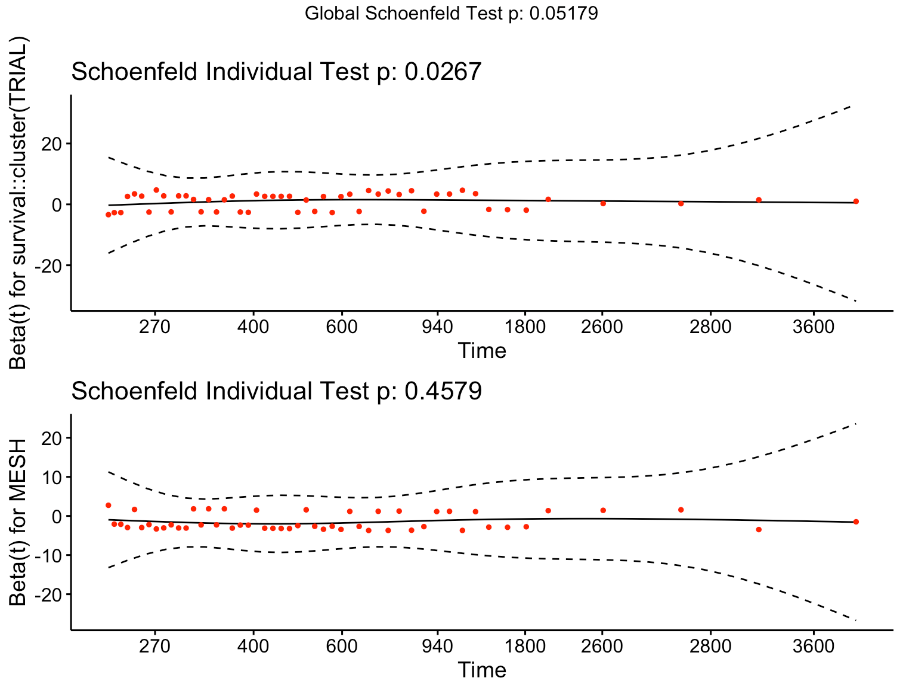

Supplement: Supplementary file 1 [file sla-283-082-s001.docx]
